# Isolation and molecular characterization of a tomato brown rugose fruit virus mutant breaking the tobamovirus resistance found in wild *Solanum* species

**DOI:** 10.1007/s00705-022-05438-2

**Published:** 2022-05-04

**Authors:** Ahmad Jewehan, Francis W. Kiemo, Nida Salem, Zoltán Tóth, Pál Salamon, Zoltán Szabó

**Affiliations:** 1 Institute of Genetics and Biotechnology, Applied Plant Genomics Group, Hungarian University of Agriculture and Life Sciences, Gödöllő, 2100 Hungary; 2 Department of Plant Protection, School of Agriculture, The University of Jordan, Amman, 11942 Jordan

## Abstract

A new tobamovirus named tomato brown rugose fruit virus (ToBRFV) overcomes the effect of the *Tm-1*, *Tm-2*, and *Tm-2*^*2*^ resistance genes introgressed from wild *Solanum* species into cultivated tomato (*Solanum lycopersicum*). Here, we report the isolation and molecular characterization of a spontaneous mutant of ToBRFV that breaks resistance in an unknown genetic background, demonstrated recently in *Solanum habrochaites* and *Solanum peruvianum*. The wild isolate ToBRFV-Tom2-Jo and the mutant ToBRFV-Tom2M-Jo were fully sequenced and compared to each other and to other ToBRFV sequences available in the NCBI GenBank database. Sequence analysis revealed five nucleotide substitutions in the ToBRFV-Tom2M-Jo genome compared to ToBRFV-Tom2-Jo. Two substitutions were located in the movement protein (MP) gene and resulted in amino acid changes in the 30-kDa MP (Phe22 → Asn and Tyr82 → Lys). These substitutions were not present in any of the previously described ToBRFV isolates. No amino acid changes were found in the 126-kDa and 183-kDa replicase proteins or the 17.5-kDa coat protein. Our data strongly suggest that breaking the newly discovered resistance in wild tomatoes is associated with one or two mutations on the MP gene of ToBRFV.

**Supplementary Information:**

The online version contains supplementary material available at 10.1007/s00705-022-05438-2.

Viruses of the genus *Tobamovirus*, such as tobacco mosaic virus (TMV) and tomato mosaic virus (ToMV), are among the most important pathogens of tomato [1–3]. To control these viruses, three resistance genes – *Tm-1*, *Tm-2*, and *Tm-2*^*2*^ – were introgressed into cultivated *Solanum lycopersicum*. *Tm-1* is an incompletely dominant gene originating from *S. habrochaites* (PI 126445) that suppresses viral infection via interactions with the helicase domain of the viral replicase (Rep) [4–6]. *Tm-2* and *Tm-2*^*2*^ were derived from *S. peruvianum* (PI 126926, PI 128650), are allelic, and confer complete dominant resistance based on a hypersensitive reaction triggered by the viral movement protein (MP) [7–10].

A newly discovered tobamovirus, tomato brown rugose fruit virus (ToBRFV), has become a dangerous pathogen by overcoming all three tobamovirus resistance genes of tomatoes [11, 12]. The sudden spread of ToBRFV to distant regions of the world [13–15] and its economic impact have prompted plant pathologists and breeders to search for new resistance sources and to incorporate resistance genes into *S. lycopersicum* cultivars. As an initial step, resistance to ToBRFV has been demonstrated in some genotypes of *S. pimpinellifolium*, *S. lycopersicum*, and *S. habrochaites* and used in breeding programs [16–19].

In the past three years, a screening program has also been started in our laboratory to search for new resistant genotypes among hundreds of wild tomato accessions representing 16 *Solanum* species [20, 21]. Following mechanical inoculation using the Jordanian ToBRFV isolate Tom2-Jo, the great majority of *Solanum* plants proved to be susceptible, but 10–50% of the individuals in nine accessions of *S. habrochaites* and a single accession of *S. peruvianum* were found to be highly resistant. The resistant plants did not express any symptoms, and the virus could not be detected, either in the inoculated leaves or in the top (systemic) leaves, by bioassays in *Nicotiana glutinosa* or using real-time polymerase chain reaction (RT-qPCR) [21]. The resistant plants were propagated vegetatively by rooting their lateral shoots in Murashige Skoog (MS) medium. After repeated inoculation, the progeny (3–5 plants of each accession) remained symptomless, except for a single plant of an *S. habrochaites* accession, which became diseased, showing mild systemic mosaic symptoms. An inoculum was prepared from this particular plant by grinding the mosaic-affected leaves in sterile 0.01 M phosphate buffer (pH 7.0, 1:5 w/v), and further inoculation was carried out on three *N. glutinosa* plants. Numerous necrotic local lesions characteristic of tobamovirus infection were observed in *N. glutinosa* leaves. Based on these results, we presumed that ToBRFV-Tom2-Jo had mutated to overcome the resistance discovered in *S. habrochaites*.

A single local-lesion isolate of the putative resistance breaker virus, named "ToBRFV-Tom2M-Jo", was propagated in *N. tabacum* cv. Samsun and characterized in comparison with its parent virus isolate, ToBRFV-Tom2-Jo. Inocula of both isolates were prepared by grinding infected tobacco leaves in phosphate buffer as described above and filtering through cheesecloth, and the extract was stored at -20°C. Leaves were inoculated by rubbing them with a glass spatula dipped into thawed virus-containing tobacco sap. Carborundum was used as an abrasive. The infectivity of each inoculum was assayed on *N. glutinosa* or *N. tabacum* cv. Xanthi-nc as local-lesion test plants. All plants were grown in an insect-proof glasshouse at 24 ± 2°C with a 14/10 h photoperiod and 50–70% relative humidity. Greenhouse and laboratory experiments were carried out under quarantine conditions.

For pathological comparison, three seedlings each of *S. lycopersicum* GCR26-Craigella (*tm-1*^CRG26^), GCR237-LA3269 (*Tm-1*), LA2088 (*Tm-2*), LA3471-Moneymaker (*Tm-2*^*2*^), and Ceglédi (*Tm*+) carrying known resistance genes were inoculated with the isolates Tom2-Jo and Tom2M-Jo. As is characteristic of ToBRFV, both isolates infected these tomato genotypes systemically, causing mosaic and leaf deformation. No phenotypic differences between the two isolates were observed. Three vegetatively propagated individuals of resistant *S. habrochaites* and *S. peruvianum* plants were then inoculated with each of the isolates. As expected, no symptoms were observed, and virus propagation could not be detected on the resistant plants inoculated with Tom2-Jo by bioassays or by RT-qPCR. In contrast, Tom2M-Jo induced mosaic symptoms on all Tom2-Jo-resistant plants, and ToBRFV was detected in their symptomatic top leaves (Fig. 1).

**Fig. 1:**
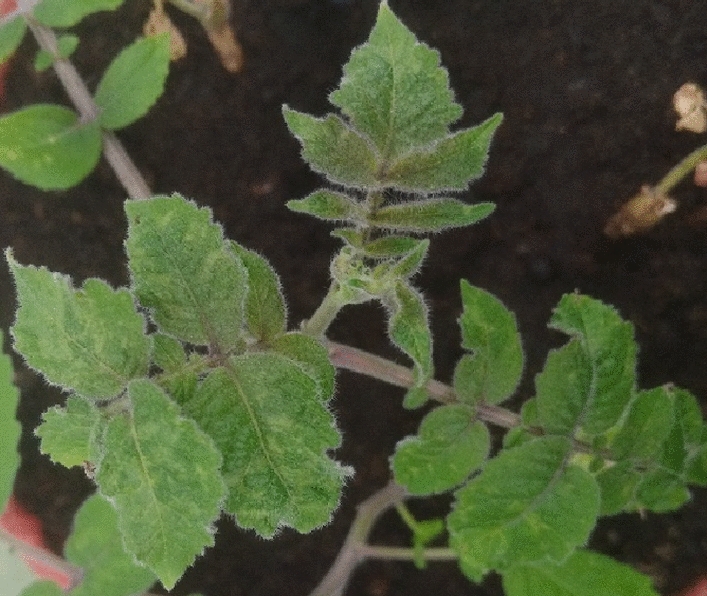
Systemic mosaic symptoms on a resistant *S. habrochaites* plant inoculated with ToBRFV isolate Tom2M-Jo

For molecular characterization, total RNA from leaves of *N. tabacum* cv. Samsun infected with ToBRFV-Tom2-Jo and ToBRFV-Tom2M-Jo, respectively, was extracted using an SV Total RNA Extraction Kit (Promega, USA) following the manufacturer’s instructions. RNA samples were used as a template for complementary DNA (cDNA) synthesis using a ToBRFV-specific primer. First-strand cDNA was synthesized using a RevertAid First Strand cDNA Synthesis Kit (Thermo Scientific, USA) kit at 42°C for 60 min, and the reaction was terminated by heating at 75°C for 5 min. Primer3 computer software (version 4.0.0) was used to design PCR primers using the ToBRFV reference genome sequence (KT383474). The whole genome of each virus (6.4-kb) was amplified from cDNA using the forward primer 5´-GTATTTTTGTTTTACAACATATACCAAC-3´ and the reverse primer 5´- TGGGCCCCTACCGGGGGTTCCGGGGGA-3´. The reaction was carried out using CloneAmp™ High Fidelity PCR Premix (Takara Bio, Japan) with denaturation at 98°C for 1 min followed by 35 cycles of 98°C for 10 s, 60°C for 30 s, and 72°C for 3 min. Amplified fragments were purified and ligated into the pJET1.2/blunt Cloning Vector using a CloneJET PCR Cloning Kit (Thermo Scientific, USA) and introduced by transformation into competent *Escherichia coli* cells according to standard protocols. The cloned fragment was sequenced using Sanger technology on an ABI Prism 3130xl Genetic Analyzer using a primer-walking strategy. The sequences of isolates ToBRFV-Tom2-Jo and ToBRFV-Tom2M-Jo were deposited in the NCBI GenBank database under accession numbers MZ323110 and MZ438228, respectively. The sequences were aligned using DNASTAR Seqman and compared using Bioedit and Multalin software with all ToBRFV genome sequences available in the NCBI GenBank database. The BLASTn, BLASTx, and BLASTp programs were used to compare the ToBRFV open reading frame (ORF) sequences and the deduced amino acid sequences of Rep, MP, and the coat protein (CP).

The complete nucleotide sequences of virus isolates Tom2-Jo and Tom2M-Jo each consist of 6,394 nucleotides and contain four open reading frames (ORFs), which is typical of tobamoviruses, including ToBRFV. Our isolates showed 99.73% sequence identity to ToBRFV-Tom1-Jo, the first Jordanian isolate of the virus [11]. Comparing the nucleotide sequences of Tom2M-Jo and Tom2-Jo, three synonymous nucleotide substitutions in the Rep region (C to T at nucleotide positions 1018 and 3622 and T to A at position 3997) and two nonsynonymous nucleotide substitutions in the MP (T to A at nucleotide positions 4975 and 5156) were detected (Fig. 2). No change occurred in the CP region. A comparison of the amino acid sequences revealed that Tom2M-Jo has no changes in Rep or CP protein but has two amino acid substitutions in the MP: Tom2-Jo has a Phe at position 22 and Asn at position 82, while the mutant Tom2M-Jo has a Tyr and Lys at the same positions (Fig. 3). Sequence alignments with 118 ToBRFV isolates [14] revealed that Tom2M-Jo has two unique nucleotide substitutions not found in other isolates, resulting in two amino acid changes. Sequence alignments with selected isolates are shown in Figs. 2, 3.

**Fig. 2:**
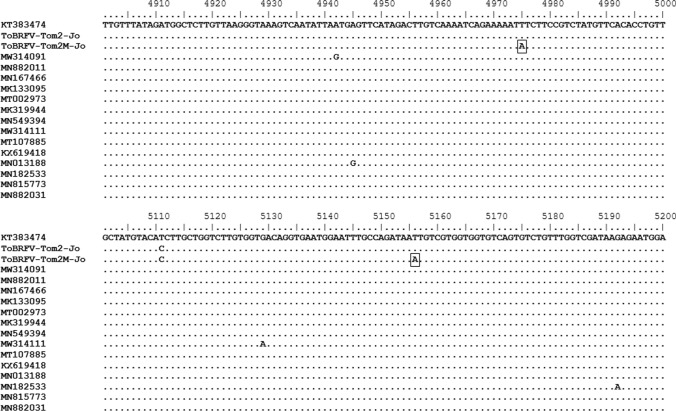
Alignment of the nucleotide sequences of the movement protein (MP) genes of ToBRFV-Tom2-Jo, Tom2M-Jo, and 15 selected ToBRFV isolates from different countries (Supplementary Table S1). Dots indicate identical nucleotides. Nucleotides that differ between ToBRFV-Tom2-Jo and ToBRFV-Tom2M-Jo are shown in a box.

**Fig. 3:**
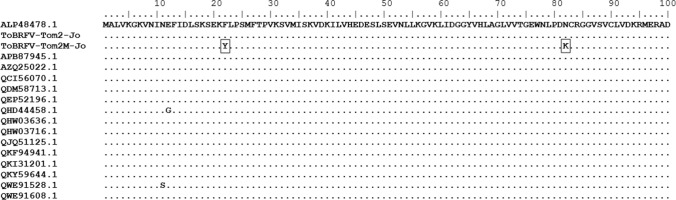
Alignment of the deduced amino acid sequences of the movement proteins (MPs) of ToBRFV-Tom2-Jo, and Tom2M-Jo, and 15 selected ToBRFV sequences from different countries (Supplementary Table S1). Dots indicate identical amino acids. The two amino acid substitutions in Tom2M-Jo are shown in a box

Soon after discovering ToBRFV, Maayan et al. [22] carried out sequence analysis to map the mutations that allowed the virus to overcome *Tm-2*^*2*^ resistance. Compared to tobamoviruses pathogenic to tomato (TMV, ToMV, and rehmannia mosaic virus [ReMV]) they pointed to twelve changes in the viral MP and nine in the Rep proteins. Recently, it was demonstrated that replacing the MP sequence of ToMV with the MP of ToBRFV resulted in a recombinant virus that overcame *Tm-2*^*2*^ resistance [23]. The key role of MP in triggering resistance was confirmed by transient expression of ToBRFV MP in resistant tomato and also in *N. benthamiana* in which the MP gene of ToBRFV and the *Tm-2*^*2*^ resistance gene of tomato were transiently co-expressed [23]. Interestingly, Yan et al. [24], using chimeric MP proteins of TMV and ToBRFV, demonstrated that six residues in the central 60–186 region of the ToBRFV MP are critical for overcoming *Tm-2*^*2*^ resistance in tomato and transgenic *N. benthamiana*.

The genetic relationships associated with the resistance in *S. pimpinellifolium*, *S. lycopersicum*, and *S. habrochaites* described by Hamelink et al. [16], Ashkenazi et al. [17], Ykema et al. [18], and Zinger et al. [19] and our resistant *S. habrochaites* and *S. peruvianum* genotypes are still unknown [21]. Consequently, it cannot be predicted whether the mutant Tom2M-Jo isolate would break the resistances discovered by the above authors. However, this study demonstrates that Tom2M-Jo is a new adaptive virus mutant breaking the strong ToBRFV resistance of wild tomatoes found recently in our work [21]. The discovery of new plant resistance traits usually stimulates research to reveal their genetic background and mechanism and prompts breeders to use the new resistances in their practice. Further investigations are needed to characterize the new ToBRFV resistance traits in *S. habrochaites* and *S. peruvianum*. Because the resistance-breaking ability of the mutant Tom2M-Jo seems to be tightly connected with change(s) within the viral MP gene, we assume that the resistance mechanism acts similarly to those directed by the *Tm-2* and *Tm-2*^*2*^ alleles. However, the resistances based on the incorporation of dominant resistance genes such as *Tm2* and *Tm-2*^*2*^ are often not durable [25, 26]. Adaptation of viruses to new resistant hosts is a well-known phenomenon, which we have witnessed in the case of ToBRFV-Tom2M-Jo, a “resistance breaker mutant of a resistance breaker virus.”

## Electronic Supplementary Material

Below is the link to the electronic supplementary material

Supplementary Material 1

Supplementary Material 2

Supplementary Material 3

## Data Availability

The data that support the findings of this study are available from the corresponding author upon reasonable request.
